# Eternal inflation, entropy bounds and the swampland

**DOI:** 10.1140/epjc/s10052-020-8412-x

**Published:** 2020-09-19

**Authors:** Ziwei Wang, Robert Brandenberger, Lavinia Heisenberg

**Affiliations:** 1 Physics Department, McGill University, Montreal, QC H3A 2T8 Canada; 2 Institute for Theoretical Physics, ETH Zurich, Wolfgang-Pauli-Strasse 27, 8093 Zurich, Switzerland

## Abstract

It has been suggested that low energy effective field theories should satisfy given conditions in order to be successfully embedded into string theory. In the case of a single canonically normalized scalar field this translates into conditions on its potential and the derivatives thereof. In this Letter we revisit small field hilltop models of eternal inflation including stochastic effects and study the compatibility of the swampland constraints with entropy considerations. We show that these stochastic inflation scenarios either violate entropy bounds or the swampland criterion on the slope of the scalar field potential. Furthermore, we illustrate that such models are faced with a graceful exit problem: any patch of space which exits the region of eternal inflation is either not large enough to explain the isotropy of the cosmic microwave background, or has a spectrum of fluctuations with an unacceptably large red tilt.

## Introduction

The inflationary scenario [1–5] is the current paradigm of early universe cosmology. In addition to explaining the homogeneity, spatial flatness and large size of our universe, the accelerated expansion of space provided by inflation yields a mechanism to explain the origin of structure in the universe [6, 7]. However, inflation is not the only scenario of early universe cosmology which is consistent with current cosmological observations. Alternatives include a bouncing cosmology with a matter-dominated phase of contraction [8], models based on Born-Infeld inspired modifications of gravity [9], the Ekpyrotic scenario [10] or an emergent cosmology with initial thermal fluctuations with holographic scaling [11], such as in *String Gas Cosmology* [12] (see e.g. [13] for a review of alternatives to cosmological inflation and [14] for alternatives of gravity theories relevant for early universe cosmology). Assuming that superstring theory is the fundamental theory which unifies all forces of Nature at a quantum level, it is interesting to ask which (if any) of the currently discussed early universe scenarios emerges from string theory. Observations indicate that our universe is entering another stage of accelerated expansion, the so-called *Dark Energy* phase. Another interesting question is how string theory might explain this phase.

Over the past thirty years there has been a lot of work attempting to realize inflation in the context of string theory (see e.g. [15] for an in-depth review). Assuming that space-time is described by General Relativity, scalar field matter is usually used in order to obtain accelerated expansion of space. However, if superstring theory yields the correct ultraviolet completion of physics at high energy scales, then there are constraints on any effective scalar field model emerging as a low energy description of physics. The criteria on an effective field theory consistent with string theory are called the *swampland* criteria (see e.g. [16, 17] for reviews). Models which do not obey these conditions are said to be in the swampland. It has been shown that these criteria severely constrain inflationary models [18] (see also [19–29]). Quintessence models [30] of Dark Energy are, at the moment, still viable [18, 28, 31–38] but will also be severely constrainable using upcoming observations [31, 32] (see also [39]).

The constraints on inflation provided by the swampland criteria have been in general obtained using the classical evolution of scalar fields during inflation. However, quantum fluctuations may have an important effect on scalar field dynamics. According to the *stochastic inflation* formalism [40], quantum fluctuations may counteract the classical force and locally drive the scalar field up the potential, i.e. to larger values of the potential energy density. This is the basis for the *eternal inflation* scenario [41, 42]. Both in the context of large field inflation [43, 44] and small field inflation [45] it has recently been studied whether these quantum effects can save inflation from the swampland constraints. In the case of large field inflation it was shown that eternal inflation can only be realized if the constant parameter which appears in the swampland constraint for a slowly rolling scalar field (see below) is much smaller than unity, and even in this case only for values of the Hubble expansion rate which are close to the Planck scale, while in the case of small field inflation occuring near a local maximum of the potential a window for eternal inflation consistent with the swampland conjectures was claimed [45].

In this Letter we study constraints on small field stochastic inflation obtained by combining the swampland constraints with entropy considerations. In analogy to the entropy of a black hole which is given by the area of the event horizon, one can associate an entropy with the Hubble horizon $H^{-1}$ (where *H* is the Hubble expansion rate) of an accelerating universe. In a phase of inflation when stochastic effects dominate the entropy associated with the event horizon decreases in regions where the scalar field moves up the potential. Using a bound on the magnitude of allowed entropy decrease from [46] we show that hilltop models of eternal inflation either violates this entropy bound, or it violates the swampland criterion on the slope of the scalar field potential. This result reinforces the conclusion that there is tension between the principles of string theory and cosmological inflation.

Attempts to reconcile small field models of hilltop eternal inflation (including stochastic effects) with the swampland criteria face another problem, the *graceful exit problem*. The density fluctuations which exit the Hubble radius during the period of inflation when stochastic effects dominate are too large in amplitude. Hence, a patch of space in which inflation comes to an end must have undergone a period of slow-roll inflation between when stochastic effects become subdominant and the end of inflation. In the case of large field inflation, the existence of such a phase is inconsistent with the swampland criteria. In the case of small field stochastic inflation [45] we must analyze the problem more carefully. Here we show that for large values of the energy density during the inflationary phase, the rolling phase in islands which exit the eternal inflation region is too short for one Hubble patch exiting the eternal inflation region to become large enough to encompass a universe of our current size. In addition, fluctuations on smaller scales are nonlinear. If the energy density in the inflationary period is lower than a given critical value, a sufficiently long period of evolution of a Hubble patch after it leaves the phase of eternal inflation can be obtained, but the resulting spectrum of fluctuations is far from scale-invariant.

While our manuscript was being completed, two papers appeared which have a large overlap with our work. A first paper [47] also demonstrated that stochastic eternal inflation is in the swampland, focusing on slightly different problems than those we concentrate on. Similar conclusions were reached in [48] which presented a detailed discussion of the Fokker–Planck equation for stochastic inflation.

## Review of the swampland criteria

We will assume that superstring theory is the correct theory of Nature. In this context, scalar fields which arise in the low-energy effective field theory of physics consist of the dilaton, moduli fields and axions. There are many scalar fields which can appear in the low energy effective action, which at first sight appears as good news for scalar field-driven inflation. However, since they all have a particular origin in string theory, their potentials and field ranges in the low energy effective field theory cannot be arbitrary.

The first condition on a scalar field $\phi$ in a low energy effective field theory description of string theory is the field range condition known as *distance conjecture* [49] which states that a particular effective field theory has a field range $\Delta \phi$ which is restricted to

1 $$\begin{aligned} \frac{\Delta \phi }{m_{pl}} \, < \, d \, , \end{aligned}$$

where *d* is a positive constant of the order 1 and $m_{pl}$ is the four space-time dimensional Planck mass. If we start at a point in field space and move a distance greater than the above one, then new string states will become low mass and have to be included in the low energy effective field theory, thus changing the theory. This condition clearly conflicts with the condition to obtain large-field inflation in canonical scalar field models of inflation since in these models the inflaton field has to move a larger distance in order to obtain a sufficient period of inflation [18]. On the other hand, since Quintessence does not require a large number of e-foldings of accelerated expansion, Quintessence models are not ruled out from the outset [18, 31, 32].

The second swampland condition [50] applies to situations where a scalar field is rolling while dominating the cosmology. It is a constraint on the slope of the potential of an effective scalar field and states that

2 $$\begin{aligned} |\frac{V'}{V}| m_{pl}\, > \, c_1 \, , \end{aligned}$$

where $c_1$ is a positive constant of order unity (the prime indicates the derivative with respect to $\phi$). This condition clearly rules out slow roll inflation models with canonical kinetic terms (models with extra friction, e.g. warm inflation [51], can be consistent with this condition [52, 53]). This condition can be derived [54] by demanding that the entropy obtained by the extra string degrees of freedom which become massless is less than the Gibbons-Hawking entropy [55] of an accelerating Hubble patch of space. It is applicable provided that the scalar field is in uniform motion, in particular during an epoch of slow roll inflation. However, it is not directly applicable if the scalar field is undergoing stochastic fluctuations without overall slow rolling.

There is a refined version of this swampland condition [54] (see also [56–69]) according to which models of effective scalar fields can be consistent with string theory even if the condition (2) is not satisfied or applicable in the region of field space where the dynamics is taking place as long as in this region

3 $$\begin{aligned} \frac{V''}{V} m_{pl}^2 \, < - c_2 \, , \end{aligned}$$

where $c_2$ is another positive constant of order unity. This condition is applicable if the scalar field starts very close to a local maximum of the potential, or if it undergoes stochastic fluctuations without net rolling. For some applications of this condition to cosmology see e.g. [23, 70–77]. Note that these conditions rule out de Sitter solutions, in particular de Sitter solutions for Dark Energy (see also [78–80] for other arguments for the inconsistency between quantum gravity and de Sitter).

Finally, effective field theories coming from string theory should also obey the *weak gravity conjecture* which states that at any point in field space, gravity is the weakest force [81].

The reason why the swampland conditions cannot be seen in the context of pure effective field theory is that at the level of an effective field theory, important string degrees of freedom associated with the string oscillatory and winding modes are not taken into account (see e.g. the detailed discussion in [17]). These modes do, indeed, play a crucial role in *String Gas Cosmology* [12], a model of a stringy early universe based on the new fundamental degrees of freedom and symmetries of string theory which are lost at the level of an effective field theory, and which yields an alternative to the inflationary paradigm of structure formation [11] (see [82] for a review and [83] for specific predictions for upcoming observations). A characteristic example of a scalar field in an effective field theory emerging from string theory is a Kähler modulus field, which in the simple setup of a toroidal compactification of the extra spatial dimension can be viewed as the radius of an extra cycle. As discussed in [84–86], the interplay of string winding and oscillatory modes leads to a minimum of the effective potential for this field which is at the string scale. This is an example of how the field distance constraint arises in a particular example. In this same example, the value of the potential at its minimum is zero, and the potential is quadratic about the minimum, thus showing that the criteria (2) and (3) are satisfied.

At first sight, it appears that in the derivation of the swampland conditions on the scalar field potential $V(\phi )$ it was assumed that $\phi$ obeys the classical equation of motion without any quantum fluctuations. However, it is known that the effective scalar field $\phi$ in any given Hubble patch obtains quantum fluctuations from inhomogeneities of larger wavelength which contribute to the local background. This gives rise to a source term in the effective equation of motion for $\phi$ whose magnitude is given by the Hubble expansion rate *H* [40]. As shown in [43, 44], inclusion of this stochastic term does not help rescue large-field eternal inflation from the swampland. In [45], however, it was suggested that the stochastic term may save small-field eternal inflation. This is the claim which we study in the following.

## Stochastic effects

In the presence of stochastic effects, the equation of motion for the effective homogeneous component of a scalar field in a given Hubble patch is

4 $$\begin{aligned} {\ddot{\phi }} + 3H {\dot{\phi }} + V' \, = \, \mathcal{N}(t, x) \, , \end{aligned}$$

and the amplitude of $\mathcal{N}$ is determined by having the stochastic term lead to a change $\langle \delta \phi ^2\rangle = \frac{H^2}{4\pi ^2}$ in one Hubble time step [40] ( finite mass corrections can be taken into accout, but it turns out that they cancel out in the condition 18 below).

The classical field variation over one Hubble expansion time $H^{-1}$ is given by

5 $$\begin{aligned} \delta \phi _c \, = \, \frac{{\dot{\phi }}}{H} \, , \end{aligned}$$

while the change in $\phi$ induced by the noise over the same time interval is

6 $$\begin{aligned} \delta \phi _q \, = \, \frac{H}{2\pi } \, . \end{aligned}$$

The given Hubble patch will remain in the eternal inflation region if the field value satisfies

7 $$\begin{aligned} | \delta \phi _q | \, > \, | \delta \phi _c |\, \end{aligned}$$

(eternal inflation itself can also occur in the case that $\delta \phi _q$ is somewhat smaller than $\delta \phi _c$ if we take into account that regions in which the scalar field moves up the potential expand faster than those in which it moves downward - see [87] for a detailed study), and if the field range where this is satisfied is greater than the size of the quantum fluctuations. Making use of the slow-roll equation of motion for $\phi$ to determine the classical movement of $\phi$ and of the Friedmann equation, coupled with the assumption that the energy density is dominated by the scalar field potential energy in order to solve for *H* in (6), the condition (7) becomes

8 $$\begin{aligned} \frac{V'}{V}m_{pl} \, < \, \frac{1}{2\pi } \frac{V^{1/2}}{m_{pl}^2}\, . \end{aligned}$$

In the range of field values where this condition is satisfied, $\phi$ undergoes stochastic fluctuations without net rolling, and hence the condition (2) is not directly applicable. In contrast, (3) can be applied.

In the case of a simple quadratic potential

9 $$\begin{aligned} V(\phi ) \, = \, \frac{1}{2} m^2 \phi ^2 \, , \end{aligned}$$

where *m* is some mass which must be much lower than the Planck mass in order that the induced cosmological fluctuations are compatible with observational bounds, the condition (8) becomes

10 $$\begin{aligned} |\phi | \, > \, \sqrt{2\pi } \bigl ( \frac{m_{pl}}{m} \bigr )^{1/2} m_{pl} \, . \end{aligned}$$

For a more general potential for large field inflation of the form

11 $$\begin{aligned} V(\phi ) \, = \, m_{pl}^4 f(\phi ) \, , \end{aligned}$$

where $f(\phi )$ is a dimensionless function, the condition (8) reads

12 $$\begin{aligned} \frac{f'}{f}m_{pl} \, < \, \frac{1}{2\pi } f^{1/2} \, . \end{aligned}$$

The condition for the scalar field potential obeying the swampland condition (2) in the field region where stochastic effects are important becomes

13 $$\begin{aligned} c_1 \,< \frac{V'}{V}m_{pl} \, < \, V^{1/2} m_{pl}^{-2} \, , \end{aligned}$$

which for values of $c_1$ of order 1 excludes inflation in field regions where effective field theory can be applied (since effective field theory breaks down when *V* approaches the Planck scale).

In the following, to be specific, we will consider the following potential

14 $$\begin{aligned} V(\phi ) \, = \, V_0 \cos (\phi / \mu ) \, , \end{aligned}$$

where $\mu$ determines the curvature of the potential, and $V_0$ its absolute value. Small field inflation takes place while $\phi$ is close to a local maximum of the potential (e.g. $\phi = 0$). When expanded about that point we get the same potential as was considered in [45]), namely

15 $$\begin{aligned} V(\phi ) \, \simeq \, V_0 \bigl ( 1 - \frac{1}{2} (\frac{\phi }{\mu })^2 \bigr ) \end{aligned}$$

(for $|\phi | \ll \mu$). The swampland condition (3) is satisfied provided that

16 $$\begin{aligned} \mu \, < \, \bigl ( \frac{1}{c_2} \bigr )^{1/2} m_{pl} \, . \end{aligned}$$

The field region where eternal inflation is possible is given by (see (8))

17 $$\begin{aligned} |\phi | \, < \, \phi _c \, \equiv \, \frac{3}{2} V_0^{1/2} \mu ^2 \, , \end{aligned}$$

(in Planck units). For eternal inflation to work, this field range needs to be larger than the size of the quantum field fluctuation. Making use of (6) this implies

18 $$\begin{aligned} \mu \, > \, \bigl ( \frac{2}{6\pi } \bigr )^{1/2} \, , \end{aligned}$$

(again in Planck units). Comparing (16) and (18) we see that for $c_2 < 6\pi$ there is a small region for $\mu$ in which both conditions can be satisfied. In the following we will assume that we are in this region of $\mu$ space.

In order to obtain a period of slow-roll inflation for $|\phi | > \phi _c$ there is an additional lower bound on $\mu$

19 $$\begin{aligned} \mu \, > \, \frac{\sqrt{2}}{3} \, , \end{aligned}$$

which can be derived by solving the slow-roll equation of motion for $\phi$ (in the approximation when *H* is treated as constant) and checking for self-consistency.

## Entropy bounds, inflation and the swampland

Another way to see the incompatibility of large field inflation and the swampland constraints is by applying entropy arguments of [46] where it was argued that the entropy decrease of any given Hubble patch cannot exceed the value one. The entropy of a local Hubble patch of an accelerating cosmology was first discussed by Gibbons and Hawking [55] and is given by the area of the Hubble horizon in Planck units, i.e.

20 $$\begin{aligned} S_{GH} \, = \, 4\pi H^{-2} m_{pl}^2 \, . \end{aligned}$$

During a time interval in a phase where stochastic effects dominate and when the scalar field can move up the potential due to quantum fluctuation, *H* increases and hence the entropy bounded by the area of the horizon decreases [46]. This process may violate the second law of thermodynamics (applied locally). It was argued [46] that, even taking quantum effect of gravity into consideration, the decreases should not be larger than $\mathcal O(1)$ in Planck units, i.e.

21 $$\begin{aligned} \delta S \, > \, -1 \, . \end{aligned}$$

Let us consider a patch in which the scalar field is moving up the potential. In this case, the field jump induced by quantum fluctuations is larger in magnitude than the jump induced by the classical force, and we can estimate the magnitude of the field jump by taking the quantum jump (6). The change in the entropy of the patch in one Hubble interval (the coherence time of the quantum fluctuations) can hence be bounded by

22 $$\begin{aligned} \delta S \, \sim \, \delta \bigl ( \frac{1}{H^2} \bigr ) m_{pl}^2 \, = \, \delta \bigl ( \frac{3 m_{pl}^4}{V}\bigr ) \, , \end{aligned}$$

where in the last step we have used the Friedmann equation. Taking the variation of the last term yields

23 $$\begin{aligned} \delta S \, \sim \, - \frac{3 m_{pl}^4}{V} \frac{|V'|}{V} \delta \phi \, . \end{aligned}$$

Considering the change of the entropy in a coherence time during which $\delta \phi = H/(2\pi )$, the entropy condition (21) then yields

24 $$\begin{aligned} \frac{|V'|}{V} m_{pl} \, < \frac{H}{m_{pl}} \, , \end{aligned}$$

which again shows that inflation at sub-Planckian densities is inconsistent with the swampland condition (2) for values of the constant $c_1$ of the order 1.

A problem with this derivation is that it is not clear why the entropy condition should be applied locally. From a global perspective there are Hubble regions where *H* will decrease, and those where *H* will increase. It is unclear how a global entropy condition should be imposed. Hence, we will turn to a more serious problem for a small-field model of inflation marginally consistent with the swampland criteria, namely the *graceful exit problem*.

## Graceful exit problem for small field eternal inflation

In the following, we recall that our goal is to show that models where small field eternal inflation is possible have a graceful exit problem.

We are interested in the dynamics of a Hubble patch which is exiting the eternal inflation regime, i.e. a region where $\phi$ is becoming larger than the value given by (7). In this regime, we can approximate the derivative of the potential by its leading term in the Taylor expansion about the origin. Our aim is to show that the period of evolution after exiting the eternal inflation region is short. Since the scalar field rapidly accelerates when it approaches $\phi = \mu$, the time interval of evolution will be short on a Hubble time scale once the field value approaches a fraction of order unity of $\mu$. Hence, for our question the use of the approximate expression for $V^{\prime }$ is justified. On the other hand, since for $\mu \sim m_{pl}$ both modes of the Klein–Gordon equation can be important, we do not drop the acceleration term. We are interested both in the effects of the second mode, and in the effects of the stochastic term in the Klein–Gordon equation in the field range where the classical force dominates.

Expressed in tems of the e-folding number *N*, the stochastic equation which we consider takes the form

25 $$\begin{aligned} H^2 \phi ''(N) + 3 H^2 \phi '(N) - V_0 \frac{\phi }{\mu ^2} \, = \, \frac{3 H^3}{2\pi } \xi (N) \, , \end{aligned}$$

where $\xi (N)$ is a Gaussian random variable with mean zero and unit variance, i.e.

26 $$\begin{aligned}<\xi (N)> \, = \, 0 \, , \,\,\,\,\, <\xi (N) \xi (N')> \, = \, \delta (N - N') \, . \end{aligned}$$

**Fig. 1:**
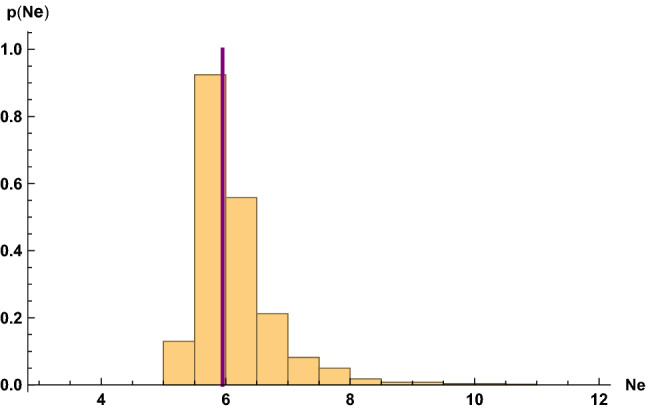
Probablity distribution of the duration of the period of slow-roll inflation based on a set of 1000 simulations with the parameters $V_0 = 10^{-8}, \mu = \frac{\sqrt{2}}{3^{1/4}}, \phi (0) = 10^{-3}$. The value of *H* was computed assuming that the potential energy dominates. The purple line indicates the value obtained neglecting the stochastic term

We performed simulations of the time evolution of the scalar field $\phi$ in the presence of the stochastic noise we have described. The parameters chosen were $V_0 = 10^{-8}$, $\mu = \sqrt{2} / 3^{1/4}$ and $\phi (0) = 10^{-3}$ (all in Planck units). By numerical solving the differential Eq. (25) with a stochastic source term in discrete time steps, we may compute the distribution of physical observables. Our results are based on a set of 1000 simulations. The results are scattered about the value which would be obtained without the stochastic term. In Fig. 1 we show the distribution of number of e-foldings until the end of inflation

**Fig. 2:**
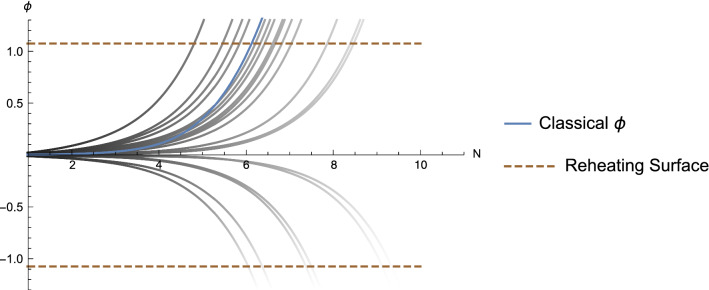
Time evolution of the scalar field once it exits the region of eternal inflation. The horizontal axis is time as measured in terms of e-folding number, the vertical axis gives the field value in Planck units. The bue curve (the one that hits the upper dashed line the fifth from the left) is the result of the analytical calculation using the slow-roll approximation. The other curves show the results of 100 numerical simulations which solve for the evolution with the same initial conditions including the stochastic terms. The horizontal dashed lines indicate the field values where inflation ends and reheating occurs. As is evident, the time duration until the field hits these surfaces is clustered about what is obtained using the analytical approximation described in the text

We can analytically estimate the period of evolution in the post-eternal phase, i.e. for $|\phi | > \phi _c$. In this phase we can neglect the stochastic term. We treat *H* as constant (thus obtaining an upper bound on the period of evolution since we are taking an upper bound on the friction coefficient). In this approximation, the equation of motion for $\phi$ has exponential solutions

27 $$\begin{aligned} \phi (t) \, \sim e^{\alpha (t - t_c)} \, , \end{aligned}$$

where $t_c$ is the initial time in this period (when $|\phi | = \phi _c$), and the two solutions for the index $\alpha$ are

28 $$\begin{aligned} \alpha _{\pm } \, = \, V_0^{1/2} \bigl [\pm \sqrt{\frac{9}{4} + 2 \frac{1}{\mu ^2}} - \frac{3}{2} \bigr ] \, . \end{aligned}$$

The dominant solution has index

29 $$\begin{aligned} \alpha _{+} \, = \, \frac{3}{2} V_0^{1/2} \gamma \, , \end{aligned}$$

where

30 $$\begin{aligned} \gamma \, = \, \bigl ( \sqrt{1 + \frac{8}{9} \frac{1}{\mu ^2}} - 1 \bigr ) \, . \end{aligned}$$

To obtain an upper bound on the duration of this phase, we can compute the time $\Delta t = t - t_c$ it takes for the field to evolve from $\phi = \phi _c$ to $\phi = \mu$, assuming that it evolves by the dominant mode. The result is

31 $$\begin{aligned} N \, \equiv \, \Delta t H \, = \, \frac{1}{\gamma } \mathrm{{ln}} \bigl ( \frac{2}{3 \mu V_0^{1/2}} \bigr ) \, , \end{aligned}$$

(where *H* is taken to be the initial value of *H*). This gives the location of the purple line in Fig. 1.

Figure 2 shows the time evolution of the scalar field once it exits the region of eternal inflation and stochastic terms are subdominant compared to the classical force in the equation of motion. The horizontal axis is time (measured in terms of *N*(*t*), the vertical axis gives the field value in Planck units. The results of 100 simulations are shown and compared to what is obtained analytically by using the slow-roll approximation described above. The initial conditions are taken to be at the field value $\varphi = \varphi _c$. Note that there are a few realizations in which the stochastic terms are strong enough to push the field back up the potential and down the other side. We see that in all cases the number of e-foldings is clustered close to the value which is obtained using the slow-roll analytical equation. The dashed horizontal lines indicate the field value where we expect the potential to turn over and reheating to occur.

We see that for values of $V_0$ similar to those which are usually used to obtain inflationary evolution (those close to the scale of Grand Unification), the period is less than $N = 50$. Hence, modes which are observed today in the microwave background and in the large-scale structure crossed the Hubble radius during the period when stochastic terms dominate the dynamics. This leads to a serious problem for the amplitude of cosmological fluctuations. The amplitude of the curvature power spectrum is given by (see e.g. [88] for a review, and [89] for a summary discussion)

32 $$\begin{aligned} P(k) \, = \, \bigl ( \frac{H^2}{2 \pi {\dot{\phi }}(t_k)} \bigr )^2 \, , \end{aligned}$$

where *k* is the wavenumber and *t*(*k*) is the time when the wavelength crosses the Hubble radius. However, during the stochastic phase this quantity is comparable or larger than unity. Note that deep in the stochastic regime, the amplitude of fluctuations is smaller than that given by the above classical value. This issue has been studied in detail in [87]. In that paper, a potential similar to the one we are using but with a value of $\mu$ one order of magnitude larger was chosen. In that case, it was found that the smaller the field value $|\phi |$, the smaller the amplitude of the fluctuations. However, the amplitude remains larger than the observed one. For our value of $\mu$ the stochastic effects on the amplitude of the power spectrum will be smaller than what was found in [87] since the quantum effects are less important relative to the classical effects compared to the situation in the model studied in [87]. Hence, the amplitude of the power spectrum, corrected by stochastic effects, will be larger than what was found in [87], and hence inconsistent with observations.Thus, our conclusion concerning the graceful exit problem is robust. The fact that the quantum corrections to the amplitude of the fluctuations is smaller in our case then in the model studied in [87] can also be seen by considering the leading order corrections to the classical power spectrum computed in Eq. (4.4) of [87]. In the case of our potential, the two correction terms are positive, and the amplitude of the enhancement term is of the order $(\mu / m_{pl})^2$ which is of the order one in our case, but much larger than one in the case studied in [87].

For values of $V_0$ small enough such that the value of *N* from (31) is larger than about 50, i.e. for

33 $$\begin{aligned} V_0^{1/2} \, < \, e^{- 50\gamma } \frac{2}{3 \mu } \, , \end{aligned}$$

the amplitude of *P*(*k*) can be made sufficiently small. However, the spectrum is not scale-invariant. It is given by

34 $$\begin{aligned} P(k) \,= & {} \, \frac{1}{\mu ^2} (3 \pi \gamma )^{-2} e^{2 \gamma N(k)} \nonumber \\= & {} \, \frac{1}{\mu ^2} (3 \pi \gamma )^{-2} \bigl ( \frac{H}{k} \bigr )^{2\gamma } \, , \end{aligned}$$

where *N*(*k*) is the number of e-foldings of evolution before the mode *k* exits the Hubble radius, and we are working in the approximation that *H* is constant (if we drop this approximation, then the spectrum will be even farther from scale-invariant). Since $\gamma$ is of the order one, the slope of the spectrum is inconsistent with observations.

Note that there is a more serious problem in the above case of small $V_0$: during the post-stochastic period of slow roll inflation the entropy criterion of [54] can be applied, and we conclude that the model lies in the swampland provided that (2) is violated, which is the case if $V_0 < 1$ in Planck units.

## Conclusions and discussion

We have considered the possibility that eternal inflation might be consistent with the swampland conditions. In the case of large field inflation we have shown using several arguments, in particular a quantum bound on the magnitude of allowed entropy decrease in a Hubble patch, that inflation is in conflict with the swampland conjectures. In the case of small field inflation there is a small window in parameter space of typical potentials such as (15) where a phase of eternal accelerated expansion could be made consistent with the swampland constraints. However, such a scenario suffers from the graceful exit problem: typically the size of the patch of the universe which exits the eternal inflation region is too small to be compatible with observations, a similar problem to the one which the original scalar field-driven model of inflation suffered from [90]. This problem can be avoided if the energy scale of the eternal inflation region is sufficiently small, as given by (33). However, in this case the induced spectrum of cosmological perturbations is red and inconsistent with observations. Furthermore, the constraint (2) can be applied during the phase of slow rolling and the model can then be shown to lie in the swamp for $V_0 < 1$.

## Data Availability

This manuscript has no associated data or the data will not be deposited. [Authors’ comment: This is a theoretical study and there are no data associated with the manuscript.]
